# The application of prenatal ultrasound in the diagnosis of congenital duodenal obstruction

**DOI:** 10.1186/s12884-020-03078-5

**Published:** 2020-07-03

**Authors:** Chan Yin, Lili Tong, Mingxiang Ma, Xiaoqun Tan, Guoliang Luo, Zhihui Fei, Dan Nie

**Affiliations:** 1 The Maternal and Child Health Hospital of Changde city, Changde, 415000 China; 2 The Maternal and Child Health Hospital of Taoyuan city, Taoyuan, 415700 China

**Keywords:** Prenatal ultrasound diagnosis, Congenital duodenal obstruction, ‘Rat tail’ sign, ‘Pliers’ sign

## Abstract

**Background:**

The purpose of this research is to summarize the prenatal ultrasound characteristics of congenital duodenal obstruction (CDO), especially in the diagnosis of duodenal diaphragm and annular pancreas. At present, few researchers have summarized the specific ultrasound features of duodenal diaphragm and annular pancreas.

**Methods:**

In this study, a retrospective analysis of 40 patients diagnosed with CDO between January 2016 and December 2019 was carried out. Data on the diagnosis, ultrasound images and outcomes of the patients were gathered, and the features of the patients were analyzed.

**Results:**

The results showed that there were 17 patients (42.5%) of congenital duodenal diaphragm, all with a ‘rat tail’ sign on the ultrasound images. Moreover, there were 4 patients (10.0%) of CDO caused by annular pancreas, all with a ‘pliers’ sign on the ultrasound images. We summarized the imaging features of the ‘rat tail’ sign and the ‘pliers’ sign.

**Conclusion:**

The main conclusion of this study was that the ‘rat tail’ sign could be used as an indirect ultrasound feature to diagnose duodenal diaphragm. The ‘pliers’ sign could be used as a direct ultrasound feature in the diagnosis of annular pancreas in CDO.

## Background

Congenital duodenal obstruction (CDO) is a common congenital malformation of the digestive tract with an incidence rate of approximately 1/2500–1/10000 [1, 2] and is caused by endogenous and exogenous factors. The endogenous factors refer to the blocking, narrowing or disconnection of the duodenum due to fetal defects in the development of the foregut. Most of the exogenous factors are caused by annular pancreas, duodenal network, volvulus, malrotation of the small intestine, intestinal atresia, rare duodenal duplication or anterior duodenal vein [3]. Duodenal diaphragm and annular pancreas are the most common endogenous factors and exogenous factors, respectively [4]. At present, there are few studies on the ultrasound features of these two kinds of obstructions in CDO.

CDO patients need to be operated on in the neonatal period after birth. The long-term survival rate has reached 86–90% with the development of CDO surgical operations in recent years [5, 6]. Previous studies have shown that early diagnosis and surgery can reduce the incidence of metabolic disorders, duodenal obstruction and intestinal failure [1, 7].

The purpose of this study is to improve the diagnostic value of prenatal ultrasound for CDO through the features of the ‘rat tail’ sign and the ‘pliers’ sign. We look forward to research results that can provide a reliable basis for clinical diagnosis.

## Methods

We reviewed 40 patients of CDO managed at the Maternal and Child Health Hospital of Changde city, China between 2016 and 2019. Data were collected through a retrospective case note study of patients with CDO detected on prenatal ultrasound scans. The data collected included maternal demographics, gestational age at the time of diagnosis, and imaging results from fetal ultrasound. Forty patients were followed up, and 5 patients were lost to follow-up. These patients were lost to follow-up because the pregnant women chose to induce labor and refused surgery, but these 5 patients could be accurately diagnosed with CDO by prenatal ultrasound. The study was approved by the Ethics Committee of the hospital.

**Fig. 1:**
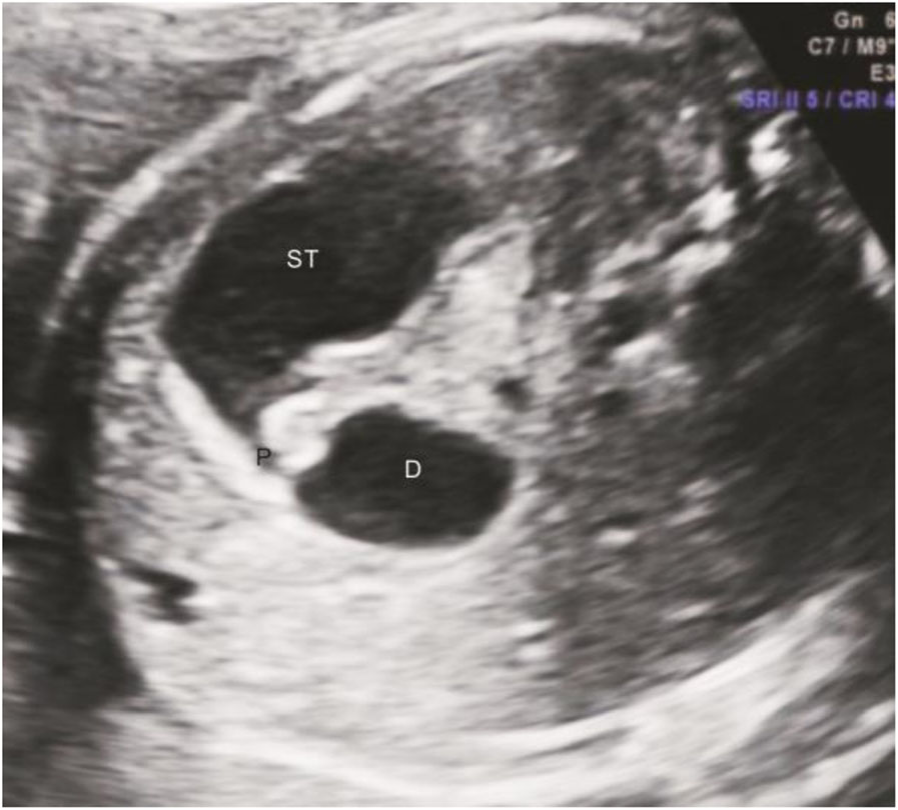
The ‘double bubble’ sign, gastric bubble and duodenal distention. Duodenum (D); Stomach (ST); Pylorus(P)

The examination equipment used in the study included GE Voluson E8, GE Voluson 730, and Philips-A70 color Doppler diagnostic ultrasound instruments. A three-dimensional abdominal volume probe with a frequency of 4–8 MHz and an abdominal detector with a frequency of 1–5 MHz were used for scanning. Ultrasound reports and images were reviewed by 4 doctors with more than 10 years of experience in prenatal ultrasound, and these doctors independently evaluated he features of CDO. The analysis mainly focused on the maximum expansion of the duodenum and amniotic fluid index. The presence of polyhydramnios was defined as an amniotic fluid index greater than 25 cm or a maximum vertical pocket exceeding 8 cm during pregnancy. At the level of the ‘double bubble’ sign, the maximum transverse diameter of the dilated duodenum (inner wall to inner wall) was measured to record the maximum dilatation of the duodenum [6]. (Fig. 1) The normal maximum transverse diameter of the duodenum should not exceed 0.2 cm at 20 to 25 weeks of gestation, 0.3 cm at 25 to 30 weeks of gestation, 0.6 cm at 30 to 35 weeks of gestation, and 0.8 cm at 35 to 40 weeks of gestation [8, 9]. Data on the neonatal outcomes were also collected, including the gestational age at birth, birth weight and associated anomalies. To evaluate the postoperative outcomes, data on the postoperative diagnosis, age at surgery, length of hospital stay, short bowel incidence and total parenteral nutrition (TPN) were recorded.

**Fig. 2:**
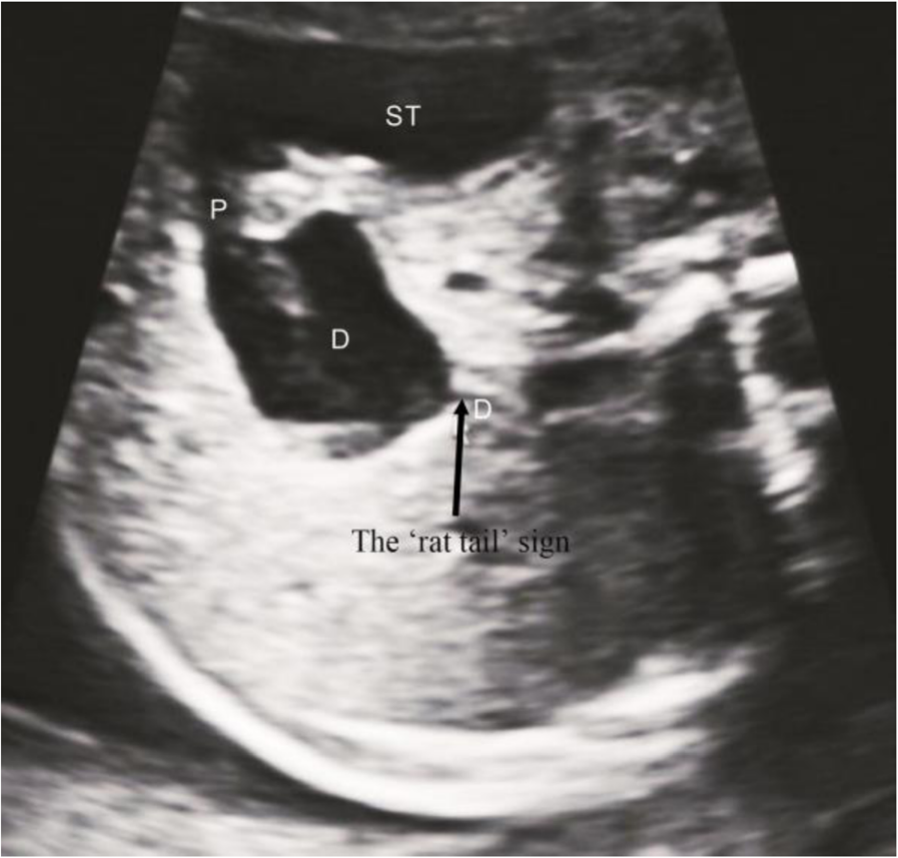
The ‘rat tail’ sign (arrow), dilated end of duodenum and connected intestine from the transverse section of upper abdomen. Duodenum (D); Stomach (ST); Pylorus(P)

Continuous scanning along the dilated end of the duodenum (the lowest part of the diaphragm) showed empty intestines with low tension. The appearance on the ultrasound image resembled a rat tail. We called this ultrasound feature the ‘rat tail’ sign, as shown in Fig. 2.

The shape of the head of the pancreas was irregular. A sharp angle was observed between the head of the pancreas and the expanded duodenum, as the bifurcated head wrapped around the ascending or descending part of the duodenum. Moreover, the expanded intestines passed through and wrapped around the pancreas. This appearance on the ultrasound image was similar to a pliers, so we called it the ‘pliers’ sign, as shown in Fig. 6a [9, 10].

All statistical analyses were performed with IBM SPSS statistical software package version 23. Cumulative results are described as proportions or percentages. A t-test was used to analyze continuous variables. Means, medians, standard deviations and ranges are used to report quantitative data. A *p*-value less than 0.05 was considered statistically significant.

## Results

From January 2016 to December 2019, 40 patients with CDO were detected by prenatal ultrasound, including 18 males and 22 females. Table 1 shows the prenatal sonographic findings of these 40 patients with CDO. The details are as follows. The average gestational age was 27.8 ± 4.1 weeks, and the average age of the pregnant women was 29 ± 5 years. The average amniotic fluid index was 18.9 ± 6.5 cm. The mean diameters of the gastric bubble and duodenum were (1.9 ± 0.7) cm and (1.1 ± 0.4) cm, respectively. Overall, 17 patients (42.5%) had only a CDO, and 23 patients (57.5%) were complicated with other malformations, including gastrointestinal tract malformations, skeletal malformations, cardiac malformations and multiple malformations.

**Table 1:** Prenatal sonographic findings in 40 patients with CDO

Characteristics	Results
Gestational age at detection (weeks)	27.8 ± 4.1
Maternal age (years)	29 ± 5
Amniotic fluid index (cm)	18.9 ± 6.5
Hydramnios (N, %)	8 (20.0)
Gastric bubble diameter (cm)	1.9 ± 0.7
Duodenum diameter (cm)	1.1 ± 0.4
Simple CDO (N, %)	17 (42.5)
With other malformations (N, %)	23 (57.5)
gastrointestinal tract malformations	13/23 (56.6)
skeletal malformations	3/23 (13.0)
cardiac malformations	3/23 (13.0)
multi-malformations	4/23 (17.4)
Down syndrome (N, %)	0 (0.0)
‘Rat tail’ sign patients^a^ (N)	16
Gastric bubble diameter (cm)	1.6 ± 0.8*
Duodenum diameter (cm)	1.0 ± 0.2*
Amniotic fluid index (cm)	20.1 ± 7.7*
‘Pliers’ sign patients (N)	4
Gastric bubble diameter (cm)	1.8 ± 0.6
Duodenum diameter (cm)	1.1 ± 0.5
Amniotic fluid index (cm)	15.6 ± 1.0

Data are presented as number (percent) and mean ± SD

^a^Data for 1 patient of missed diagnosis was included

*Comparison between the ‘rat tail’ sign patients and the ‘pliers’ sign patients, *p* < 0.05

Forty patients of CDO showed a ‘double bubble’ sign, of which 38 patients of the ‘double bubble’ sign appeared in middle pregnancy, and 2 patients of the ‘double bubble’ sign were still present in the third trimester. Among them, 1 patient of the ‘double bubble’ sign caused by midgut volvulus still existed at the second examination during the third trimester. The other patient of the ‘double bubble’ sign disappeared at the second examination during the third trimester as the amniotic fluid index continued to increase.

Among the 40 patients, 21 patients were diagnosed with duodenal diaphragm based on the ‘rat tail’ sign, of whom 16 patients were confirmed by surgery and anatomy, 5 patients were misdiagnosed. 1 patient was missed. Five patients with annular pancreas were diagnosed according to the ‘pliers’ sign, of whom 4 patients were confirmed by surgery and anatomy, 1 patient was misdiagnosed. 1 patient was missed.

Eight patients were accompanied by polyhydramnios, all of which occurred in the third trimester. There were 3 patients of polyhydramnios in CDO with the ‘rat tail’ sign and 0 patients of polyhydramnios in CDO with the ‘pliers’ sign. There was no difference in the amniotic fluid index or the diameters of the gastric bubble and duodenum between the patients with the ‘rat tail’ sign and those with the ‘pliers’ sign (Table 1).

Down syndrome was not found in these 40 patients, but 1 patient had abnormal single nucleotide polymorphism (SNP) array test results. According to the characteristics of the ultrasound image and the location of the obstruction, we considered that the abnormality was caused by annular pancreas. After the operation, it was confirmed that the abnormality was posterior annular pancreas. We also tested the family and neonatal genes, and the results showed that no abnormality was found from the parents’ examination. One rare copy number variation (CNV) was found in the neonate. It was seq [GRCh37]dup (7)(p22.1) chr7:g4949476-5188260dup, with a fragment size of 0.24 Mb. According to the standards and guidelines for the interpretation of sequence variants by The American College of Medical Genetics and Genomics (ACMG), dup (7)(p22.1) was evaluated as a CNV of unknown clinical significance.

The fetal outcomes of the 40 fetuses with CDO included voluntary termination of the pregnancy (30 patients), surgery (8 patients), fetal death in the abdomen (1 patient) and postnatal death (1 patient). The fetal death rate in utero was 2.5% (1 of 40). This fetus died in utero at 34 weeks and had duodenal diaphragm. Moreover, the postnatal mortality rate was 2.5% (1 of 40). This patient with normal chromosomes was born at 38 weeks with duodenal diaphragm and diaphragmatic hernia and died on the third day. All fetuses, except for 5 who were lost to follow-up, were confirmed to have CDO by surgery (8 patients) or autopsy (27 patients). Twelve patients (34.3%) had only CDO. Twenty-three patients (65.7%) were complicated with other malformations. The prenatal diagnosis of the 5 patients lost to follow-up was simple CDO without other malformations.

The postoperative outcomes are also presented in Table 2. Eight neonates underwent surgery. The average age at surgery and the length of hospital stay were (4.3 ± 1.0) and (21.6 ± 4.7) days, respectively. The incidence of short bowel was 0, and all 8 neonates needed TPN.

**Table 2:** The postoperative outcomes

Characteristics	Results
Fetal Outcomes	
VTOP(N/%)	30 (75.0)
Surgery(N/%)	8 (20.0)
Fetal death in utero	1 (2.5)
Postnatal death	1 (2.5)
Diagnosis after birth	
Simple CDO (N, %)	12/35 (34.3)
With other malformations (N, %)	23/35 (65.7)
gastrointestinal tract malformations	13/23 (56.6)
skeletal malformations	3/23 (13.0)
cardiac malformations	3/23 (13.0)
multi-malformations	4/23 (17.4)
Gestational age at birth (weeks) (*N* = 9)	37.6 ± 1.4
birth weight (kg) (*N* = 9)	2.9 ± 0.5
age at surgery (days) (*N* = 8)	4.3 ± 1.0
Neonatal length of hospital stay (days) (*N* = 8)	21.6 ± 4.7
Short bowel incidence (*N* = 8)	0 (0)
Need for TPN (*N* = 8) (N, %)	8/8 (100%)

Note: *VTOP* voluntary termination of pregnancy, *TPN* total parenteral nutrition

## Discussion

CDO was caused by endogenous and exogenous factors, of which duodenal diaphragm was the most common endogenous factor and annular pancreas was the most common exogenous factor [4]. There were 17 patients of duodenal diaphragm and 4 patients of annular pancreas in this study. This was the key of our analysis.

In this study, 21 patients with duodenal diaphragm were found, of whom 16 patients were confirmed by operation and anatomy, 5 patients were misdiagnosed. 1 patient was missed. The imaging feature of the ‘rat tail’ sign was found in all 17 patients. The specific analysis is shown below.

**Fig. 3:**
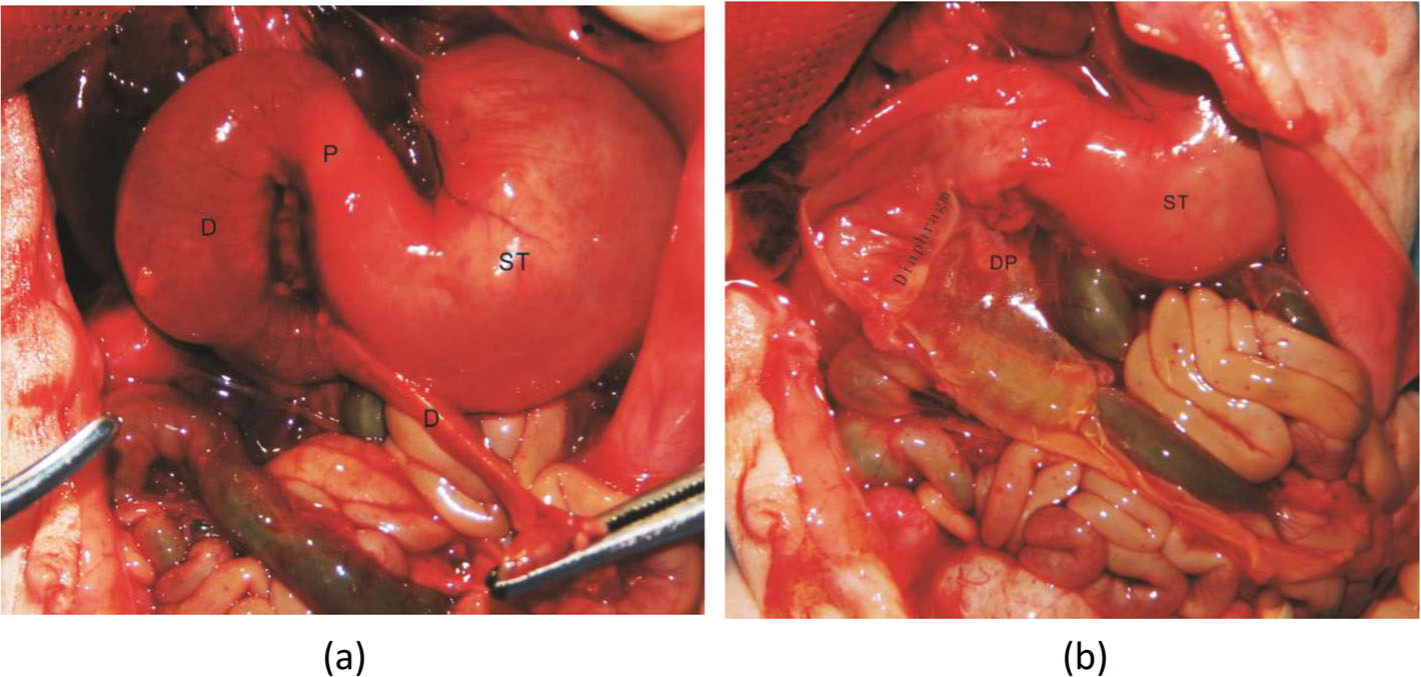
The diaphragm in the anatomy of the patient. (**a**) Outside view (**b**) Internal view. Duodenum (D); Stomach (ST); Pylorus(P)

The diaphragm is a thin and transparent membrane structure that cannot be directly detected by ultrasound. However, it can be indirectly diagnosed by the ‘rat tail’ sign. The ‘rat tail’ sign was formed by the dilated intestine above the diaphragm and the empty intestine below the diaphragm. The diaphragm could occur in any part of the duodenum, especially near the ampulla of Vater. We found the diaphragm based on the anatomy of the patient, as shown in Fig. 3.

**Fig. 4:**
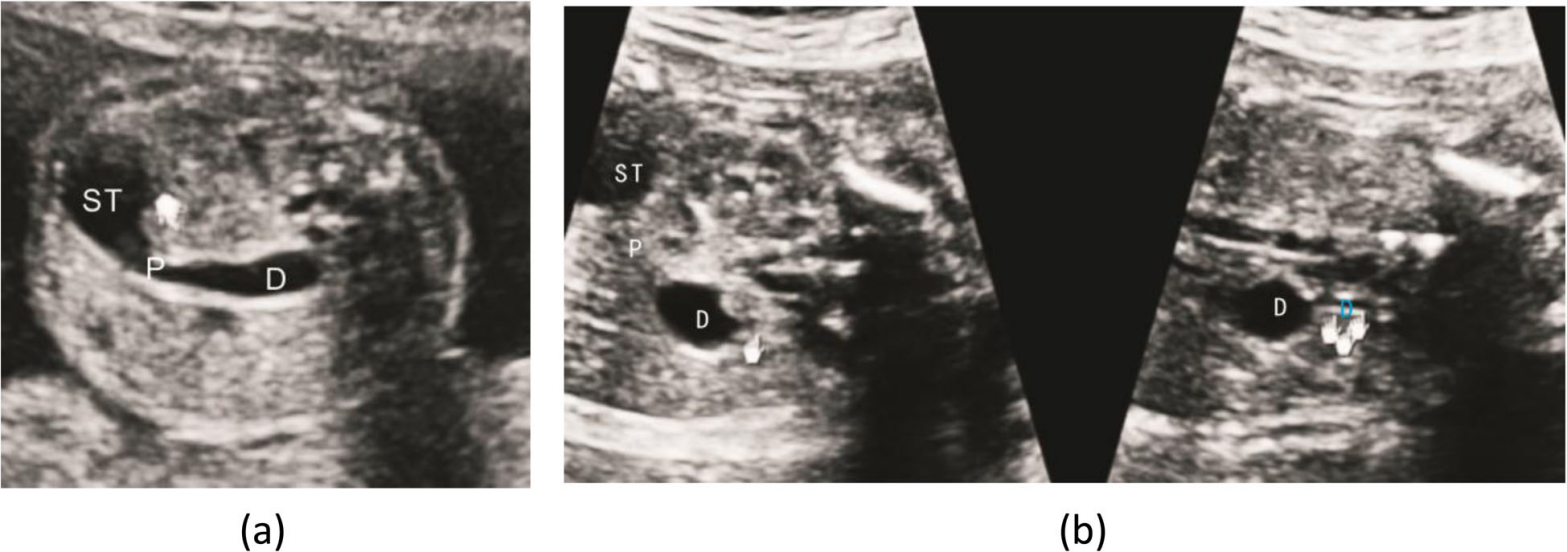
**a** The duodenum shows a ‘wind bag’ shape from the transverse of upper abdominal. **b** The end of the ‘wind bag’ sign shows a ‘rat tail’ sign from transverse of upper abdominal and fetal oblique coronal. Duodenum (D); Stomach (ST); Pylorus(P)

When the diaphragm was large, the stricture of the distal intestine led to the expansion of the duodenum above the diaphragm, showing a ‘wind bag’ shape. The intestine with little tension under the diaphragm presented a ‘rat tail’ sign, as shown in Fig. 4.

**Fig. 5:**
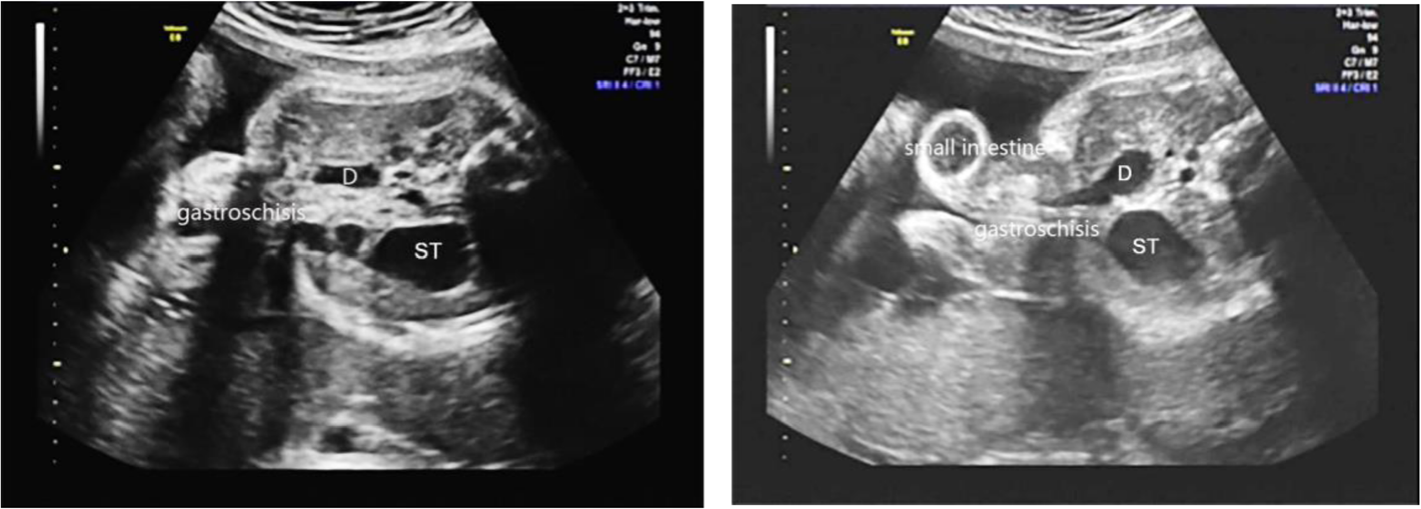
The position of gastric bubble, duodenum and jejunum ileum change dynamically with the change of abdominal pressure. Duodenum (D); Stomach (ST)

Misdiagnosis and missed diagnosis were divided into the following three patients: (1) the dilated duodenum was near the porta hepatis: during the scanning process, the portal hepatic duct was mistakenly considered the empty intestinal duct with a continuous duodenal obstruction, which appeared similar to the ‘rat tail’ sign; color Doppler ultrasonography (CDFI) was helpful for the differential diagnosis of this condition, and dynamic observations could also improve the accuracy of this diagnosis; (2) when the diaphragm was large, it restricted the small intestine under the duodenojejunal flexure and caused dilation of the duodenum and part of the small intestine; jejunal obstruction was easy to diagnose due to the position of intestinal distention behind the duodenum; and (3) when CDO was combined with diaphragmatic hernia or gastroschisis, the position of the intestine was abnormal. In the patient of diaphragmatic hernia, the gastric bubble was located in the thoracic cavity, and the duodenum was located in the abdominal cavity. Ultrasound could easily lead to a misdiagnosis due to peristalsis of the gastrointestinal tract and the presence of a diaphragm. In patients with gastroschisis, the locations of the gastric bubble, duodenum and jejunum and ileum changed dynamically with abdominal pressure. The diameter and shape of duodenal dilatation changed greatly, as shown in Fig. 5. This was also easy to misdiagnose.

The ultrasound characteristics of CDO in the prenatal diagnosis of annular pancreas have rarely been reported in previous studies. Some researchers used three-dimensional ultrasound imaging technology to diagnose annular pancreas. Moreover, other researchers used two-dimensional ultrasound to observe the size and shape of a normal fetal pancreas to diagnose annular pancreas [4]. However, there have been no relevant reports on the diagnostic method and ultrasound imaging characteristics of fetal annular pancreas. It was difficult to diagnose annular pancreas because of the different developmental stages of the pancreas in different gestational weeks. Annular pancreas is a congenital developmental deformity. Approximately 6 weeks after the embryo develops, with translocation of the duodenum, the ventral pancreas is also transposed to the posterior inferior portion of the back pancreas. In the the seventh week, the pancreas and the abdominal pancreas begin to contact and merge into one pancreas at last, and the two pancreatic ducts also fuse with each other. Therefore, one theory holds that annular pancreas arises because the ventral duodenal primordium did not fuse with the dorsal primordium as the duodenum rotates. The other theory holds that annular pancreas is because the ventral and dorsal primordium of the pancreas were hypertrophied at the same time. The second segment of the duodenum was completely or partially surrounded, resulting in obstruction [11].

In this study, 5 patients with annular pancreas were diagnosed by the ‘pliers’ sign, of whom 4 patients were confirmed by operation, 1 patient was misdiagnosed. 1 patient was missed. The specific analysis is shown below.

**Fig. 6:**
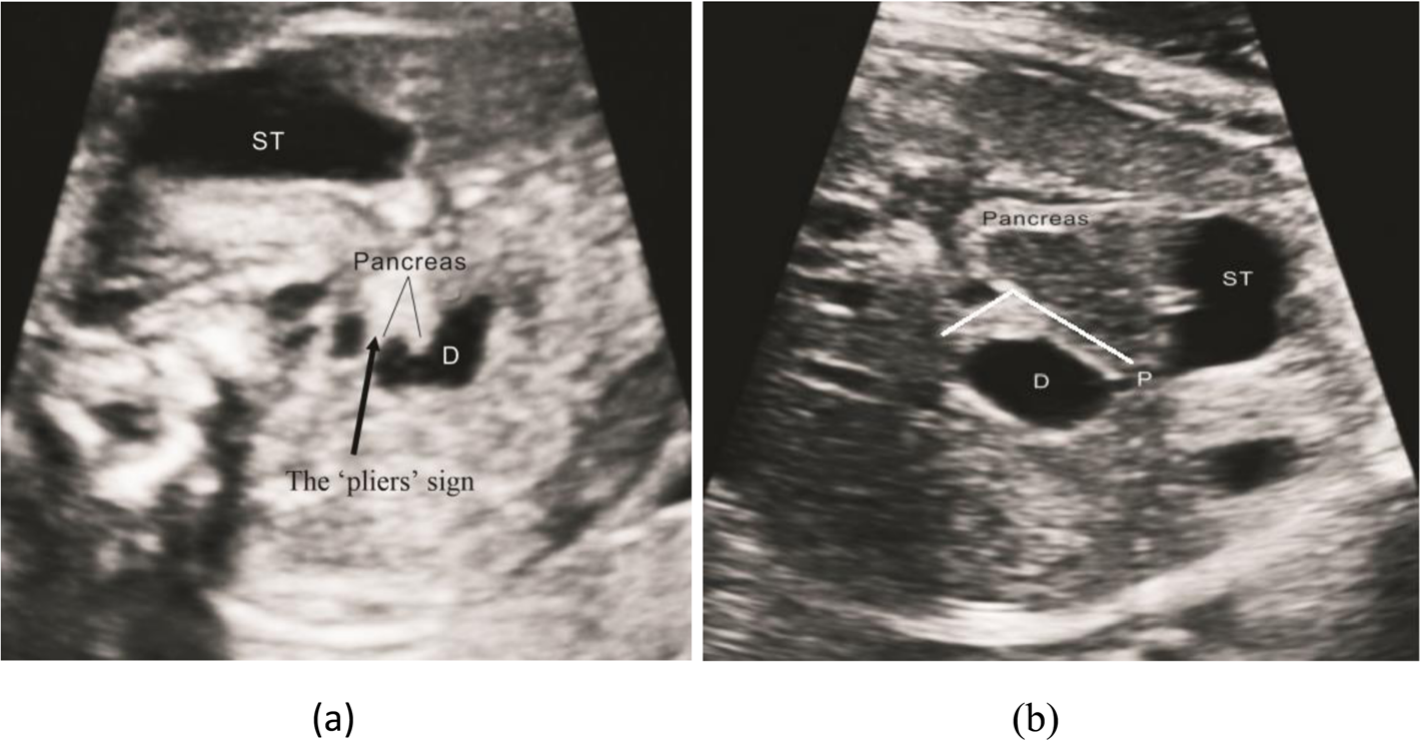
**a** The bifurcation of the head of pancreas is characterized by ‘pliers’ sign. The head of pancreas is hypertrophic and irregular; **b**. A patient of misdiagnosed duodenal dilatation with ‘rat tail’ sign. The shape of pancreas is regular. The angle between pancreas and duodenum is obtuse. Duodenum (D); Stomach (ST); Pylorus(P)

**Fig. 7:**
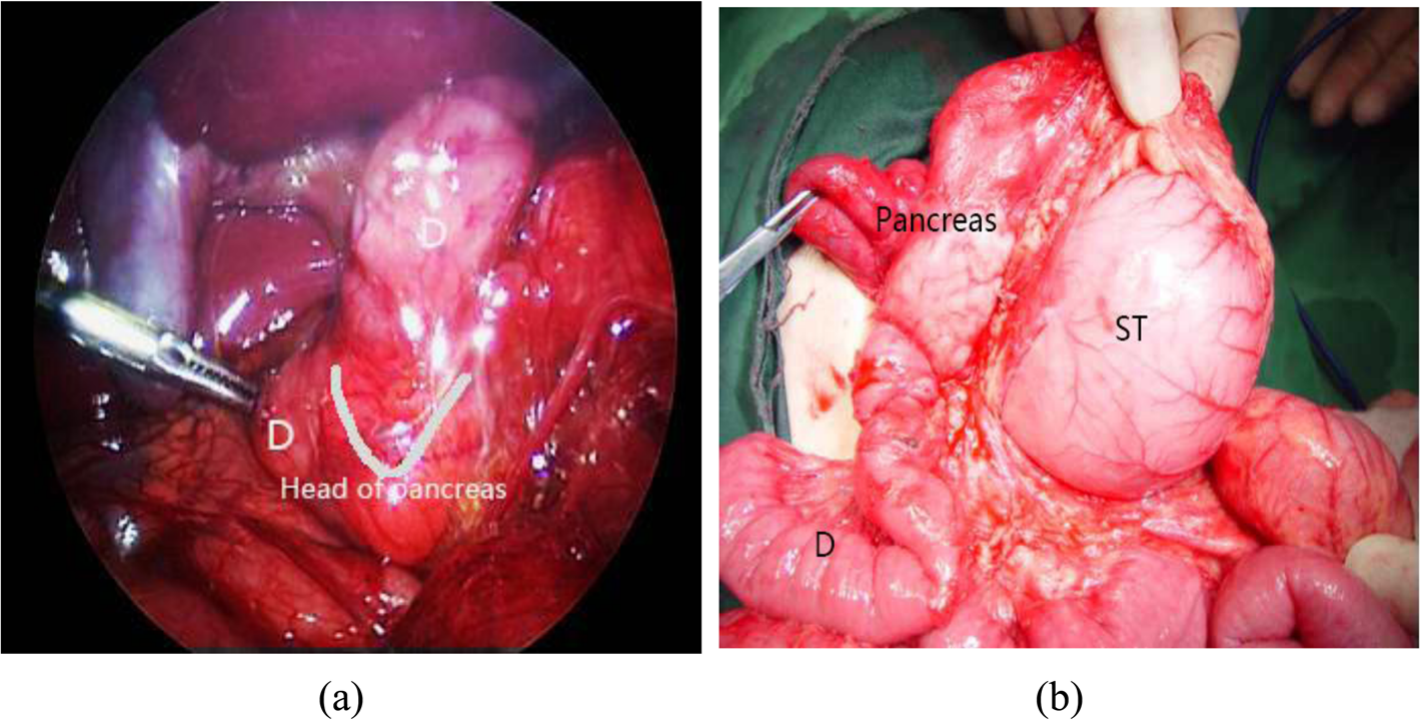
**a** Laparoscopic annular pancreas.; **b** Annular pancreas in open vision. Duodenum (D); Stomach (ST)

In CDO caused by annular pancreas, we found that there were common features on prenatal ultrasound images. The head of the pancreas was hypertrophied and irregular in shape. The bifurcation of the head of the pancreas was characterized by the ‘pliers’ sign, wrapping the ascending and descending parts of the duodenum at an acute angle [12]. The structure of the head of the pancreas and the wall of the duodenum were irregular, and the boundary was not clear. Moreover, the head of the pancreas and the dilated duodenum were intertwined (Fig. 6a). However, in duodenal obstruction caused by other reasons, the structure of the pancreas and head of the pancreas were regular, but the structure of the duodenal wall was irregular, as shown in Fig. 6b. Even if the pancreas and head of the pancreas were close to the wall of the pancreas and intestine, the boundary between the pancreas and head of the pancreas was clear, and there was no ‘pliers’ sign showing intertwining. By analyzing the causes of the unclear structure of the head of the pancreas and the duodenal wall, some experts found that the fat gap between the annular pancreatic tissue and the duodenal intestinal wall tissue disappeared because of their interweaving [9]. The anatomy of annular pancreas is shown in Fig. 7. It was confirmed that the diagnosis based on prenatal ultrasound was accurate.

One patient misdiagnosed by operation was shown to have duodenal diaphragm. In a retrospective study of this image, it was found that the angle of the ‘pliers’ sign was obtuse rather than acute. The strongly echogenic wall of the intestine was mistaken for the bifurcated head of the pancreas surrounding the descending part of the duodenum. The structure of the expanded duodenal wall was regular, and the serous layer of the duodenum was intact. Because the expanded duodenum compressed the head of the pancreas, the head of the pancreas was close to the wall of the duodenum. However, the boundary between the two was clear, and the shape of the pancreas was regular, as shown in Fig. 6b.

The missed diagnosis occurred in a 34-week gestation age fetus. The shape and structure of the pancreas could not be clearly displayed. The image could not accurately reflect the ‘pliers’ sign as an obstruction. The reason for the missed diagnosis was that there was a certain relationship between the appearance of the pancreas and gestational age. It has been reported that the fetal pancreas can be identified by ultrasound after 20 weeks of gestation. The highest display rate was 80.0% at 20–23 weeks of gestation and 38.4% at 36–39 weeks of gestation [13, 14].

**Fig. 8:**
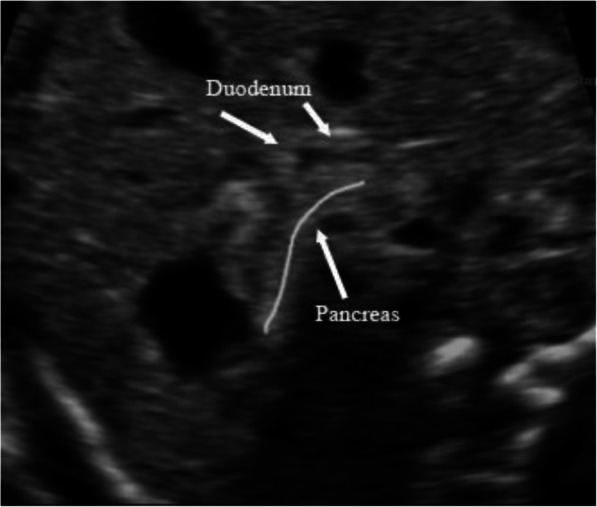
Normal ultrasound images of duodenum and pancreas

Figure 8 shows a normal duodenum and pancreas. There is no dilatation of the duodenum, and the pancreas is adjacent to the duodenum. It is clear that there is no ‘rat tail’ sign or ‘pliers’ sign.

It is also worth noting that the incidence rate of Down syndrome was 0 out of 40 patients of CDO, which was obviously different from that in other studies [6, 15, 16]. A possible reason is that prenatal screening is well performed in China. The pregnancy would be terminated if Down syndrome is found in the examination. There were also studies that showed that Down syndrome did not affect the incidence rate of duodenal obstruction [6].

In one patient, there was no ‘double bubble’ sign at 24 weeks of gestation, and the amniotic fluid index was 21 cm. At 32 weeks, the ‘double bubble’ sign appeared, and the amniotic fluid index was 28 cm, and the patient was diagnosed with duodenal diaphragm. After 36 weeks, the ‘double bubble’ sign disappeared, and the diameter of the duodenum returned to normal with an amniotic fluid index of 29 cm. No abnormality was found in the follow-up after birth. This suggests that duodenal dilatation may be transient and can later be restored to a normal dimension [17]. It may also be that the thin diaphragm ruptured spontaneously due to pressure. The obstruction disappeared, and the inner diameter returned to normal.

## Conclusions

This study mainly focused on the ultrasound characteristics of CDO and reached the following conclusions:

(1) The ‘rat tail’ sign could be used as an indirect ultrasound feature in the diagnosis of congenital duodenal diaphragm;

(2) The ‘pliers’ sign could be used as a direct ultrasound feature in the diagnosis of CDO caused by annular pancreas;

(3) Down syndrome did not affect the incidence rate of duodenal obstruction.

However, the clinical diagnosis of duodenal diaphragm and annular pancreas by using the ultrasound characteristics of the ‘rat tail’ sign and the ‘pliers’ sign require the operator to have a deep understanding of these two diseases and the instrument to have a high resolution. Regarding the other causes of CDO, this study could not draw a conclusion because of the limited samples.

## Data Availability

The data analysed during this study are included in the tables in this published article. The datasets used during the current study are available from the corresponding author on reasonable request.

## Abbreviations

CDO: Congenital duodenal obstruction
D: Duodenum
ST: Stomach
P: Pylorus
SNP: Single nucleotide polymorphism
CNV: Copy number variation
VTOP: Voluntary termination of pregnancy
TPN: Total parenteral nutrition
